# Interview with Dr Philip McCarthy

**DOI:** 10.2217/ijh-2017-0022

**Published:** 2018-02-22

**Authors:** Philip McCarthy

**Affiliations:** 1 Department of Medicine, Blood & Marrow Transplant, Roswell Park Comprehensive Cancer Center, Buffalo, NY, USA

**Keywords:** autologous and allogeneic hematopoietic stem cell transplant, graft-versus-host disease, multiple myeloma, cellular therapies

## Abstract

**Philip McCarthy speaks to Roshaine Wijayatunga, Senior Editor:** Oncology Philip McCarthy completed his MD at Tufts University School of Medicine, Boston, MA, USA and his Internal Medicine Residency at Yale, New Haven Hospital, New Haven, CT, USA. His Fellowship was completed at Brigham and Women’s Hospital and the Dana Farber Cancer Insitute, Harvard University, Boston, MA, USA. His research interests are devoted to developing novel intensive and reduced intensity allogeneic (allo) and autologous (auto) hematopoietic stem cell transplant (HSCT) approaches for the treatment of hematologic disorders, leading to improved patient outcomes and decreased toxicity. He has over 20 years of experience treating HSCT patients and directing clinical and translational HSCT research studies. He has served as chair or co-chair of several clinical trials including CALGB 100104, a Phase III clinical trial evaluating lenalidomide maintenance after auto-HSCT for multiple myeloma (MM). This study demonstrated an improved progression-free and overall survival for MM patients receiving lenalidomide maintenance therapy after auto-HSCT. The Roswell Park Comprehensive Cancer Center Blood and Marrow Transplant team has developed a systematic approach to the evaluation and treatment of HSCT patients with a specific focus on predicting and minimizing treatment-related mortality. The team participates with a core group of basic science and clinical researchers who are committed to the investigation of the complications of auto- and allo-HSCT and to the developing novel approaches to improve outcomes.

## Could you please provide a brief summary of your career up to this point?

I went to Tufts University School of Medicine, followed by internship and residency training at Yale New Haven Hospital and fellowship training at Brigham and Women’s Hospital and Dana Farber Cancer Institute. I became an instructor in medicine at Brigham and Women’s Hospital, moved to Baylor College of Medicine as an Assistant Professor of Medicine for 5 years. I moved to Roswell Park Comprehensive Cancer Center (RPCCC) in 1996 where I currently am a Professor of Oncology and Internal Medicine at RPCCC and the State University of NY at Buffalo. I am Director of the RPCCC Blood and Marrow Transplant program. My research interests are devoted to developing novel intensive and reduced intensity allogeneic (allo) and autologous (auto) hematopoietic stem cell transplant (HSCT) approaches for the treatment of hematologic disorders, leading to improved patient outcomes and decreased toxicity. I have over 20 years of experience treating HSCT patients and directing clinical and translational HSCT research studies. I have served as chair or co-chair of several clinical trials including CALGB 100104, a Phase III clinical trial evaluating lenalidomide maintenance after auto-HSCT for multiple myeloma (MM). This study demonstrated an improved progression free and overall survival for MM patients receiving lenalidomide maintenance therapy after auto-HSCT. We have developed a systematic approach to the evaluation and treatment of RPCCC HSCT patients with a specific focus on predicting and minimizing treatment-related mortality.

## You are currently director of the Blood & Marrow Transplant program at the Roswell Park Comprehensive Cancer Center. What is your assessment on the current state of bone marrow transplants & how they have developed over the past decade?

I use both terms: blood and marrow transplant and HSCT. We use mobilized peripheral blood, bone marrow and cord blood as hematopoietic stem cell sources for auto- and allo-transplant. Over time we have seen a major decrease in the morbidity and mortality of auto- and allo-transplant for the treatment and cure of selected hematologic malignancies. Some of the newer treatments are the use of immunotherapy such as chimeric antigen receptor (CAR) T-cell therapy, natural killer (NK) cell therapy and half-matched (haploidentical) HSCTs. These exciting new therapies are accompanied by new challenges such as cytokine-release syndrome, persistence and or loss of the effector cells and persistence or loss of the anti-tumor effect.

## Graft-versus-host disease is a complication of transplantation that occurs both acutely & chronically: can you tell us about some of the latest developments in treating this condition?

Graft-versus-host disease (GvHD) has remained a major complication of allo-HSCT. It is accompanied by a graft-versus-tumor (GvT) effect, primarily against hematologic malignancies such as leukemia and lymphoma. A major research interest is examining strategies to diminish the morbidity and mortality of GvHD and preserve and enhance the GvT effect. Some of the new therapies are the use of post-transplant cyclophosphamide to diminish alloreactivity and decrease GvHD severity, Janus kinase inhibition and Bruton's tyrosine kinase inhibition for GvHD therapy, selective T-cell depletion and T-cell add back for diminishing GvHD and enhancing GvT.

## What are some of the key aspects to consider when developing better strategies for bone marrow transplantations for patients?

Decreasing toxicities and improving efficacy!

## What obstacles would you say present the most significant problems with regard to improving outcomes for patients undergoing bone marrow transplants?

We need to develop more effective regimens for auto- and allo-HSCT. We need to incorporate more effective strategies to control disease during the HSCT and maintenance strategies to prevent disease relapse and progression.

## Conversely, what are some of the most recent breakthroughs that have advanced the transplantation field?

As mentioned earlier, the use of post-transplant cyclophosphamide has increased the pool of potential donors (haploidentical donors) and the use of cellular therapy: natural killer cells, CAR-T cells and other novel strategies such as bridges to allo-HSCT or for the treatment of relapse after auto- or allo-HSCT.

## What is your research in MM focusing on at present, & could you tell us about some of your upcoming work?

We are looking to develop new strategies to control MM in the long term. These include novel maintenance strategies to prolong response and decrease relapse. We also need to develop early end points for that are surrogates for long term outcome. As novel and better therapies for MM are developed, patients are living longer and trials need to stay open longer to determine for progression free and overall survival endpoints, thus an earlier endpoint such as eradication of minimal residual disease from the bone marrow may serve as a surrogate for long term outcome. Since trials will stay open longer, can we utilize early end points as surrogates for outcome. Can the complete response rate at a fixed time after primary therapy (6 months, 1 and 2 years) or minimal residual disease absence in the bone marrow serve as an early end point for long-term outcome? In addition, we are looking at the immune profile of MM patients undergoing therapy. We have found certain immune cells are correlated with disease outcome. For example, higher gamma delta T-cell numbers are associated with improved progression free and overall survival. Are these epiphenomena or can we develop strategies to increase γδ T cells and improve outcome?

## What is being done to better improve the management of this malignancy?

There are a plethora of new drugs and new approaches to the treatment of MM. These include two monoclonal antibodies (elotuzumab [elo] and daratumumab [dara]), histone deacetylation inhibition as well as new proteasome inhibitors (carfilzomib and ixazomib) and immunomodulatory drugs (pomalidomide), all approved within the last few years for MM therapy. We need to develop new approaches to decrease toxicity and improve efficacy for our patients. A big issue is the cost of these drugs. We need to work with clinicians, pharmaceutical companies, regulatory agencies, insurance companies and our government to provide the optimal care for MM patients.

## How has the field changed with the emergence of immunotherapies in the last few years?

We are just beginning to understand how to use Elo and Dara as well as beginning to incorporate them in upfront induction therapy. Checkpoint inhibition with immunomodulatory drugs was found to be toxic for MM patients so we need to carefully evaluate new therapies for upfront and relapsed/refractory MM treatment. Cellular therapies such as CAR-T cells with targets such as B-cell maturation antigen are in early Phase I studies.

## What are your expectations for the future of the field?

I expect to see more agents for the treatment of MM, especially with novel mechanisms of action. The future is bright for our patients as we look for the best approach to cure this disease.

## Closing statement

I am very fortunate to be a clinical investigator in the field of auto- and allo-HSCT and in the field of MM therapy. I look forward to the day when we can cure all patients of not only hematologic cancers but all cancers.

